# Ovarian teratoma leading to colonic intussusception: a case report

**DOI:** 10.3389/fonc.2025.1555443

**Published:** 2025-05-01

**Authors:** Xiujuan Qiu, Jiahao Gao, Jun Gao

**Affiliations:** ^1^ Department of Oncology, Xiangyang No.1 People’s Hospital, Hubei University of Medicine, Xiangyang, China; ^2^ Department of General Surgery, Xiangyang No.1 People’s Hospital, Hubei University of Medicine, Xiangyang, China

**Keywords:** intussusception, intestinal obstruction, case report, surgical treatment, ovarian teratomas

## Abstract

Intussusception in adults is relatively rare compared to that in children and is typically caused by underlying benign or malignant intestinal diseases. We report a case of a 40-year-old female who presented with colonic intussusception and obstruction caused by an ovarian teratoma invading a segment of the colon. Contrast-enhanced CT imaging revealed a soft tissue mass within the colon, and colonoscopy identified a large cystic lesion approximately 6.0 × 6.0 cm in size located 20 cm from the anal verge. This lesion obstructed the lumen, causing significant narrowing. Preoperatively, the condition was misdiagnosed as intussusception caused by a colonic diverticulum. During laparoscopic exploration, a left ovarian tumor invading the colon was identified as the cause of the intussusception and obstruction. Consequently, partial colectomy and left oophorectomy were performed. Histopathological examination confirmed the diagnosis of a mature ovarian teratoma. The postoperative recovery was uneventful, and the patient was discharged in good health. This case report presents a rare instance of intussusception, highlighting the challenges associated with achieving an accurate preoperative diagnosis. Surgical intervention remains the primary treatment modality for patients with this condition.

## Introduction

Intussusception in adults is relatively rare, accounting for less than 5% of all intussusception cases and approximately 1% of intestinal obstructions (1). It is characterized by a segment of the intestine and its associated mesentery (the intussusceptum) telescoping into an adjacent segment (the intussuscipiens), resulting in luminal obstruction. Adult intussusception is clinically uncommon, typically secondary, and often presents with non-specific symptoms. About 90% of adult cases arise from benign or malignant tumors, inflammatory lesions, or Meckel’s diverticulum (2). Consequently, surgical resection remains the primary treatment modality for adults with intussusception.

Ovarian teratomas arise from the aberrant proliferation and aggregation of germ cells within the ovary. Because germ cells are pluripotent, these tumors can contain a wide array of human tissues, including hair, sebaceous material, skin, and teeth. Teratomas are classified as either mature (benign) or immature (malignant). Although many teratomas remain asymptomatic, large lesions can lead to symptoms such as abdominal distension, abdominal pain, and pressure effects on surrounding organs (3).

A comprehensive review of both national and international literature revealed no documented cases in which an ovarian teratoma in a woman penetrated the intestinal wall to form a large polypoid mass resulting in intussusception. Here, we present a case of colonic intussusception caused by an ovarian teratoma in an adult patient treated at our hospital. The detailed case history follows.

## Case presentation

The patient, a 47-year-old woman, was admitted to the hospital on September 3, 2024, presenting with a chief complaint of lumbar and abdominal distension and discomfort lasting one day.

### Past medical history

The patient reported a previously good state of health, with no history of diabetes, hypertension, coronary artery disease, or pancreatitis. She also denied any history of smoking, alcohol use, or prior surgeries.

Physical Examination upon Admission:Vital Signs: Temperature: 36.2°C, Pulse: 80 beats/min, Respiratory Rate: 20 breaths/min, Blood Pressure: 126/87 mmHg. The patient was conscious and alert, with no cyanosis of the lips or enlargement of superficial lymph nodes. No pharyngeal congestion; bilateral tonsils were not enlarged; no jugular vein distension was noted. Bilateral breath sounds were clear, with no dry or wet rales. Percussion revealed resonant lung fields. Heart rate was 85 beats/min with a regular rhythm and no pathological murmurs. The abdomen was flat, without visible intestinal peristalsis, and soft on palpation. The liver and spleen were not palpable below the costal margin. Murphy’s sign was negative. There was mild rebound tenderness in the left abdomen but no shifting dullness. Bowel sounds were weak. No percussion pain was elicited in the bilateral renal areas. No edema was observed in the lower extremities.

White Blood Cell (WBC) Count: 12.65 × 10^9/L (elevated; reference range: 4–10 × 10^9/L)Neutrophil (NEUT) Percentage: 87.2% (elevated; reference range: 40%–75%).Tumor marker testing preoperatively:CEA 1.8ng/ml, CA199 12.2IU/ml, CA72-4 0.36IU/ml, CA50 5.97IU/ml, CA24 25.8IU/ml, all within normal range.

**Figure 1:**
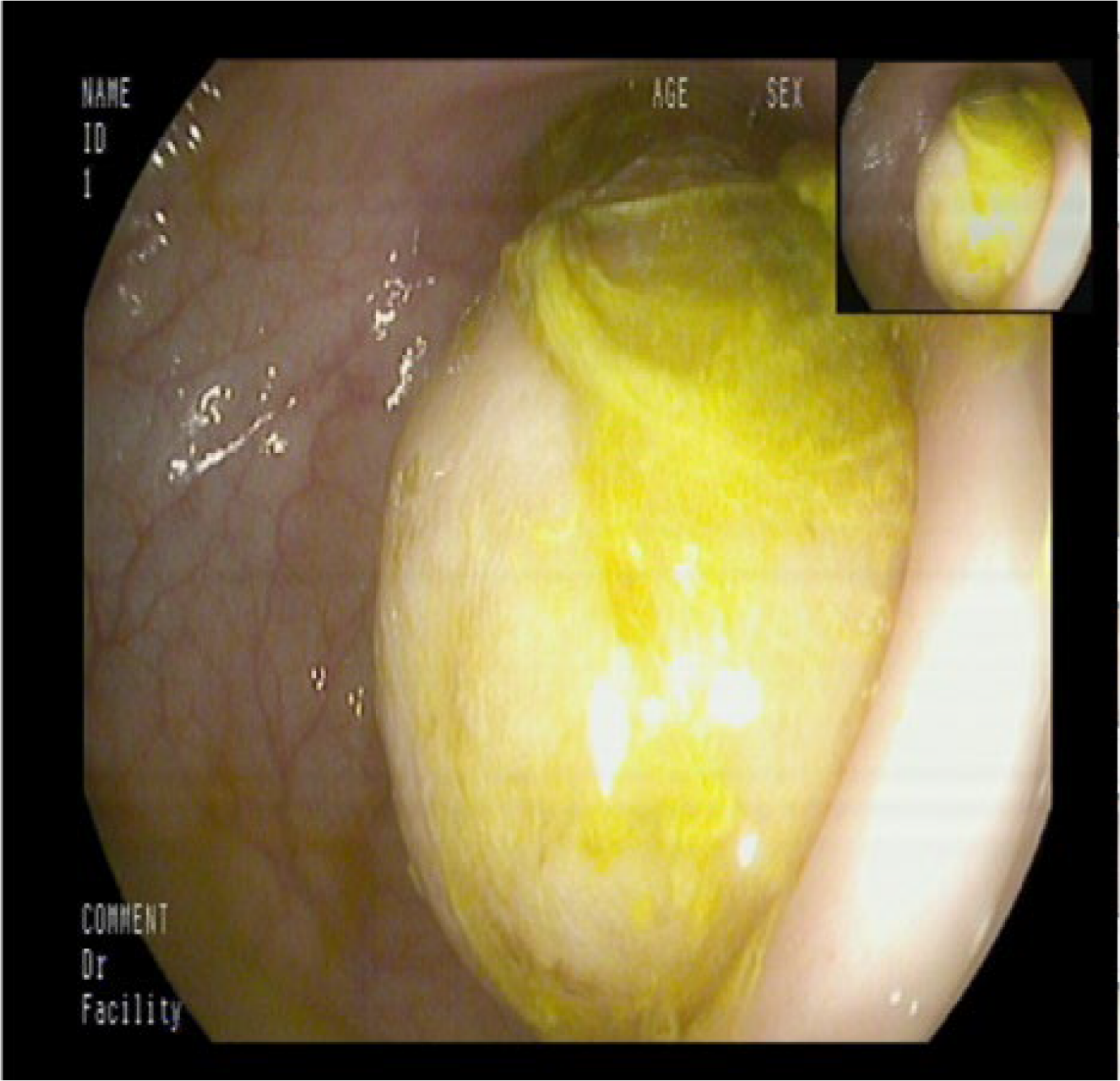
The colonoscopy image shows a large mass within the intestinal lumen.

Approximately 20 cm from the anal verge, a diverticulum-like opening measuring about 0.8 cm was identified. Adjacent to this opening, nodular hyperplasia was noted. Below the opening, a large cystic lesion measuring approximately 6.0 × 6.0 cm was observed, obstructing the lumen (**Figure 1**). The lesion had a smooth surface, resulting in marked luminal narrowing. The diagnosis considered included intussusception or a large diverticulum with fecalith formation. As a benign lesion was suspected, no biopsy was taken.

**Figure 2:**
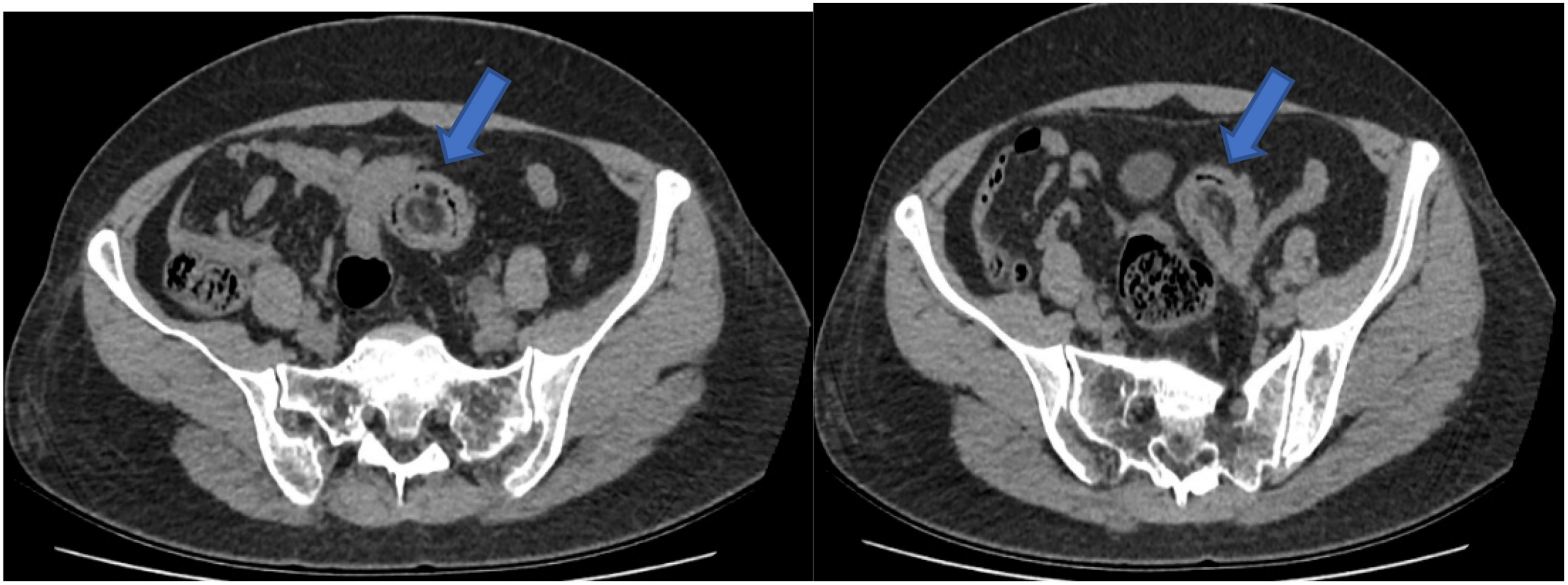
Axial slice of computed tomography showing intussusception (arrow).

Abdominal CT Findings: a mass was identified in the lower left abdomen, although its nature remains undetermined. Additionally, left-sided ureteral dilation and hydronephrosis were noted, warranting further investigation to clarify the underlying cause (**Figure 2**).

**Figure 3:**
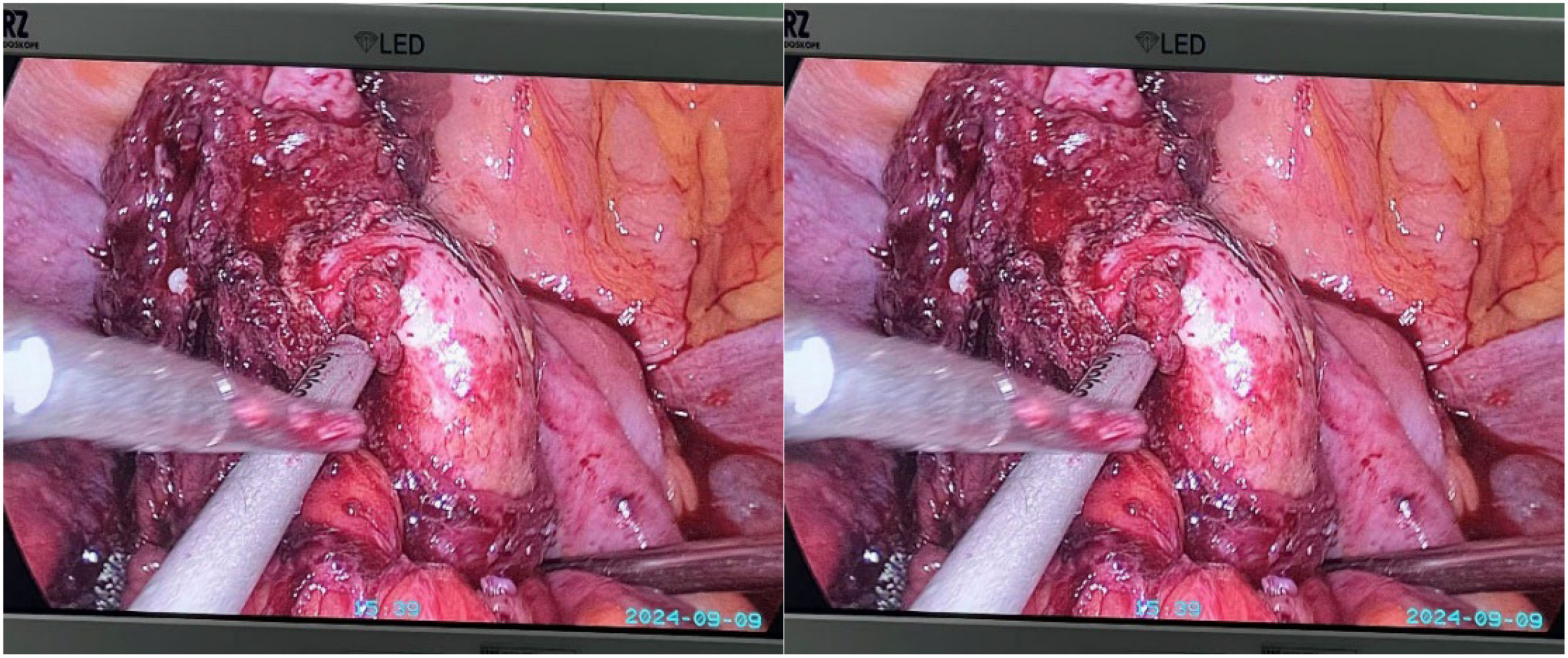
Intraoperative images.

Under general anesthesia, laparoscopic exploration was performed. Intraoperative findings revealed a mass in the left adnexa tightly adherent to the sigmoid colon, with the mass invaginating into the sigmoid colon (**Figure 3**). Consequently, a laparoscopic left adnexectomy and partial sigmoidectomy were performed. Further exploration revealed marked dilation of the left ureter, with the pelvic segment appearing rigid and narrowed over approximately 2 cm due to its adhesion to the ovarian tumor. Dense adhesions around the left ureter were excised, allowing the ureter to be fully mobilized and exposed. A tubular stapler was inserted through the anus to perform an end-to-end sigmoid colon-rectum anastomosis. Since preoperative bowel preparation was performed, a stoma was not created during the surgery. A 5 cm incision was made in the mid-lower abdomen to extract the specimen.

**Figure 4:**
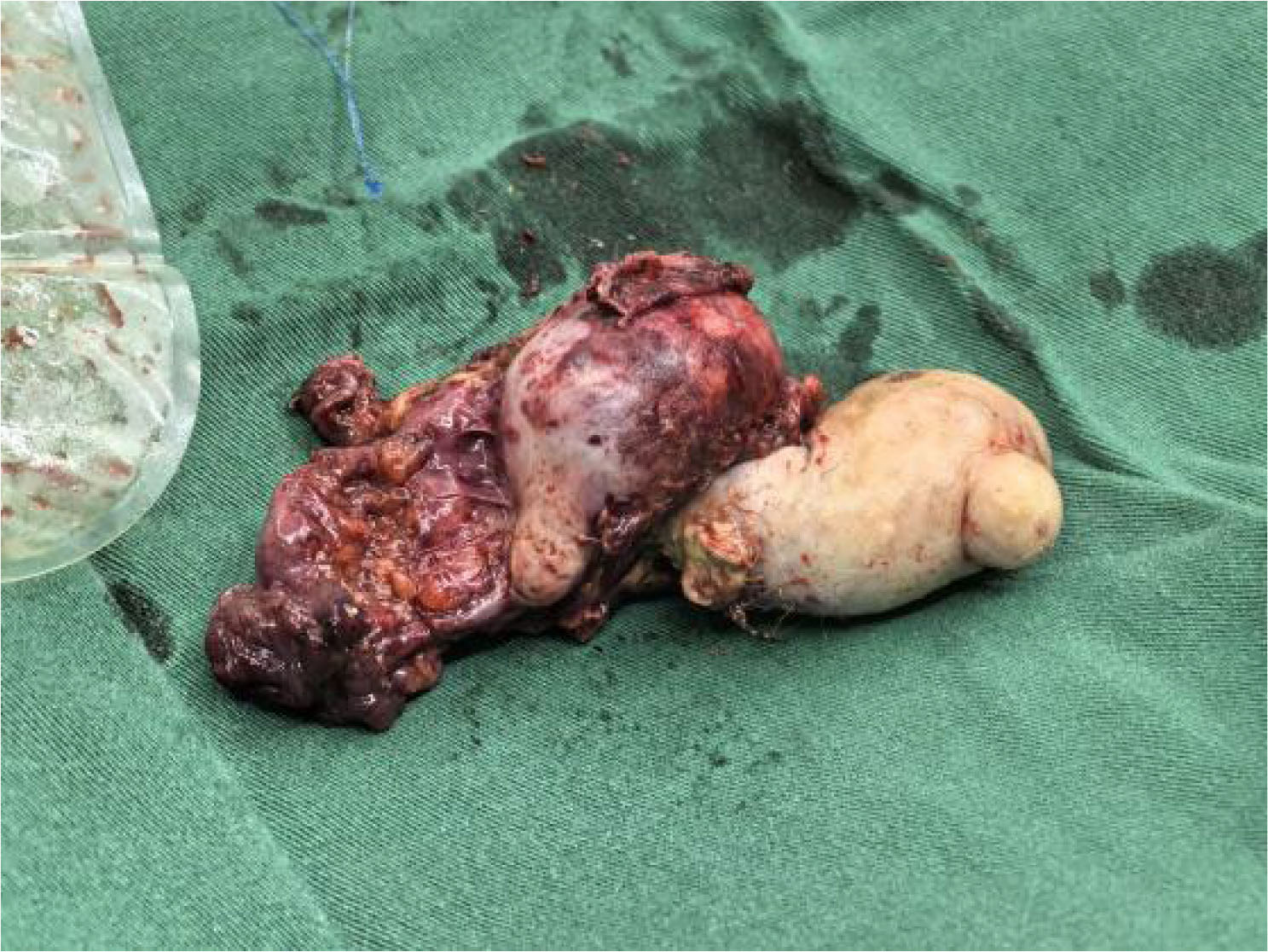
Postoperative tissue specimens.

**Figure 5:**
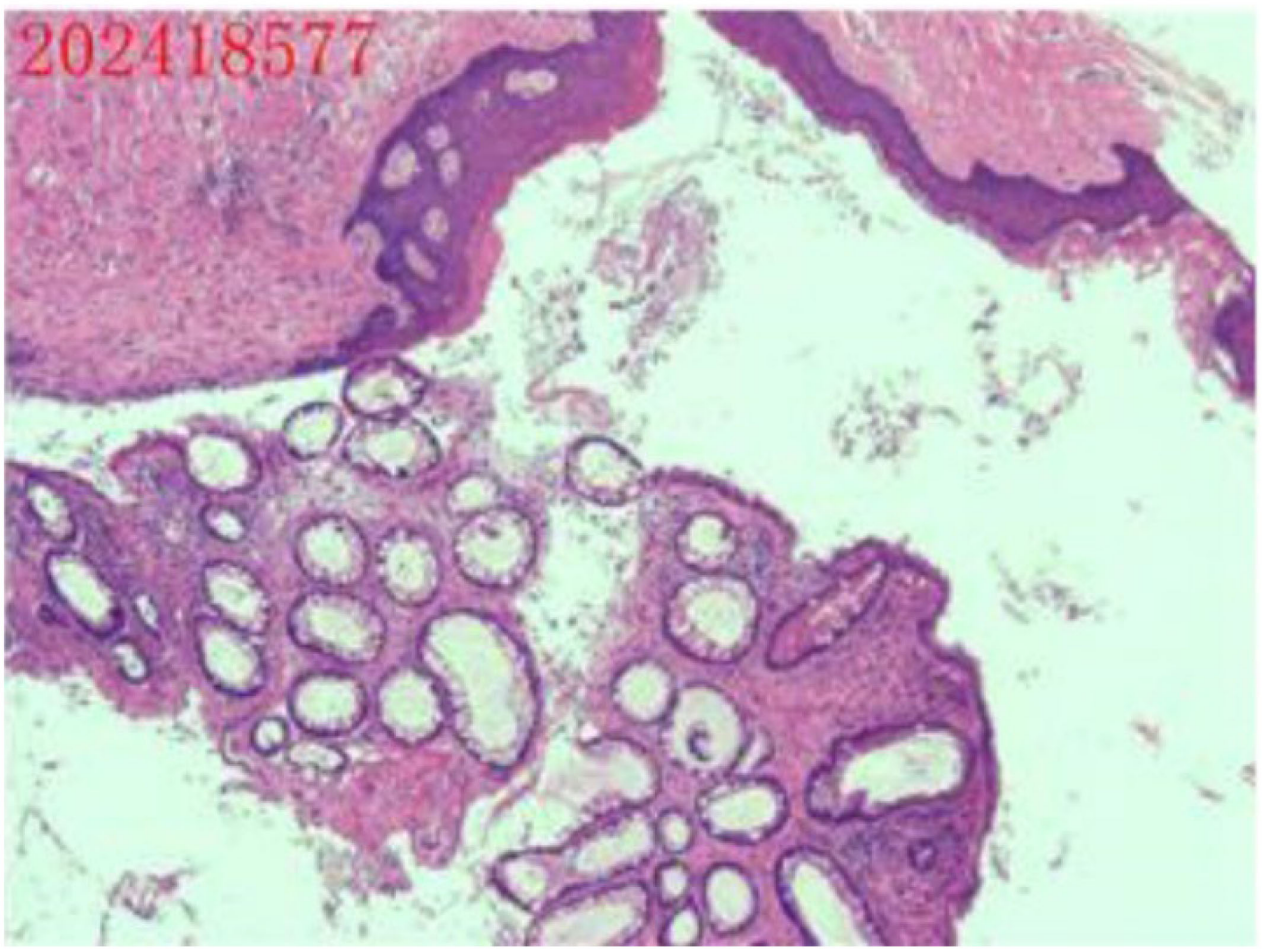
Postoperative pathology report. The ovarian mass was diagnosed as a cystic mature teratoma.

Gross Appearance of the Left Adnexal Specimen: an irregular, gray-red ovarian tumor measuring 7 cm × 6 cm × 3.5 cm was identified. A 7 cm × 4 cm × 3.5 cm gray-red nodule was observed at one end of the mass. The cut surface displayed both cystic and solid components, containing hair, sebaceous material, and bone (**Figures 4**, **5**).

Under the guidance of an enhanced recovery after surgery (ERAS) protocol, the patient began carbohydrate intake on the first postoperative day and was discharged on the seventh postoperative day. One month later, she underwent ureteral stent removal. Follow-up to date indicates good recovery with no reported complications.

## Discussion

Intussusception is most frequently observed in children, the relatively high mobility of the ileocecal region contributes to the increased incidence; these are generally not associated with underlying organic lesions. By contrast, adult intussusception accounts for roughly 5% of all cases and is therefore considered rare in clinical practice (4–6). According to the literature, 90% of adult intussusception cases can be attributed to an identifiable pathological cause (7), most frequently tumors, polyps, inflammation, diverticula, adhesions, or intraluminal foreign bodies. The primary mechanism involves a lesion in the intestinal wall or lumen that disrupts normal peristalsis, allowing forceful contractions of the proximal segment to push the affected portion of the intestine into the adjacent distal segment, resulting in intussusception (8–10).

Multi-slice spiral CT is considered the most sensitive imaging modality for detecting intussusception (11). Abdominal CT scans are unaffected by intestinal gas and provide a diagnostic accuracy ranging from 78% to 100%, offering a more reliable method for detailed evaluation and localization (12). Doppler ultrasound is particularly valuable especially relevant for pediatric and pregnant patients. According to Amr et al. (13), abdominal ultrasound can achieve a diagnostic accuracy exceeding 75%. Colonoscopy also plays an important role by allowing direct visualization and biopsy of the underlying lesion. Wang et al. (14) recommend performing colonoscopy either preoperatively or intraoperatively in cases of colonic intussusception to clarify the nature of the lesion and guide surgical decision-making. In the present case, colonoscopy identified a mass within the intestinal lumen; however, due to limited experience, it was initially misdiagnosed as a colonic diverticulum. Intraoperative exploration later confirmed that the mass was caused by an ovarian tumor invading the colon.

Ovarian teratoma is a common ovarian tumor arising from germ cells, containing components derived from two or three germ layers. Many cases are asymptomatic and are incidentally detected during routine physical examinations or imaging studies (15). Symptoms of ovarian teratoma can include abdominal pain, abdominal distension, and irregular menstruation. As the tumor enlarges, it may exert pressure on adjacent organs, resulting in abdominal discomfort, nausea, vomiting, or constipation. In severe cases, complications such as intestinal or bladder fistula formation, and even malignant transformation with metastasis, may occur. Preoperative diagnosis typically relies on ultrasound, MRI, or CT imaging.

In this case, the patient was initially misdiagnosed with intussusception caused by a large colonic diverticulum, based on colonoscopic findings, abdominal CT results, and clinical symptoms. The correct diagnosis—intussusception caused by a teratoma—was only confirmed during intraoperative exploration. Reasons for the Misdiagnosis: 1. Common Causes of Intussusception Intussusception is typically associated with intestinal lesions, such as tumors or diverticula, which guided the initial diagnostic considerations. 2. Chronic Invasive Growth of the Mature Ovarian Teratoma: The slow, infiltrative expansion of the teratoma obscured its boundaries, making it difficult to identify as the actual cause of intussusception. 3. Over-Reliance on Colonoscopy Diagnostic efforts relied heavily on colonoscopic findings, and no preoperative ultrasound examination was performed. An ultrasound might have provided additional information about the mass’s origin and nature.

In this case, the ovarian teratoma was pathologically confirmed as a mature teratoma. Remarkably, it penetrated the intestinal wall and grew in a rod-like fashion, ultimately causing intussusception—an exceedingly rare presentation. A previously reported instance involved a pediatric testicular teratoma in the abdominal cavity that invaded the colonic wall, forming a large mass responsible for intussusception (16). Possible Mechanisms of Tumor Invasion 1. Anatomical Proximity: The left ovary is situated adjacent to the lateral side of the sigmoid colon, allowing the tumor to exert direct pressure on the colonic wall. 2. Germ Cell Origin and Embryonic Development As a germ cell–derived tumor, a teratoma can contain normal embryonic layers. These may align with the corresponding embryonic layers of the colon, potentially facilitating tumor invasion into the colonic wall, leading to chronic perforation and eventual rupture into the intestinal lumen. Only a few reports have documented mature ovarian teratomas invading the rectum (17, 18). One hypothesis suggests that teratoma rupture allows slow leakage of sebaceous material into the abdominal cavity, causing peritoneal irritation and granulomatous peritonitis. Repeated inflammatory stimulation may in turn result in chronic colonic perforation and tumor invasion. In the present case, the patient experienced preoperative left ureteral narrowing leading to ureteral dilation and hydronephrosis. Postoperative histopathology confirmed that chronic inflammatory adhesions around the ureter were responsible for the narrowing. Surgical dissection of these adhesions relieved the ureteral obstruction. Additionally, postoperative pathology revealed hyaline degeneration in surrounding fibrous, adipose, nerve, and smooth muscle tissues, as well as amyloid deposits, further indicating the presence of earlier inflammatory processes in the abdominal cavity.

Common symptoms of intussusception include abdominal pain, nausea, vomiting, abdominal distension, and tenderness, most of which are nonspecific. In this case, the patient initially presented with discomfort in the left lumbar region, seeking medical attention for presumed ureteral obstruction. Subsequent CT imaging revealed a mass within the colon. The intussusception did not cause intestinal obstruction, so the patient did not exhibit abdominal distension, visible bowel loops, or other signs of intestinal obstruction. Management of symptomatic or complicated ovarian mature cystic teratomas typically requires surgical intervention (18). In the authors’ view, laparoscopic exploration offers substantial benefits over open surgery. Laparoscopy enables precise identification of both the cause and the location of the lesion, after which a small incision can be made to enter the abdominal cavity. The affected segment of the intestine can then be externalized for the necessary surgical procedures. This approach prevents the challenges associated with poorly positioned or overly large incisions in open surgery, ultimately enhancing patient outcomes and recovery.

In conclusion, adult intussusception typically arises from an underlying organic lesion and often presents with nonspecific clinical symptoms. Abdominal CT is recommended as the first-line diagnostic modality, and once the diagnosis is confirmed, timely surgical intervention is advised. Laparoscopic exploration should be pursued initially, followed by a surgical plan tailored to intraoperative findings. For tumors of uncertain character, intraoperative frozen-section examination is recommended to clarify the pathology and guide selection of the most appropriate surgical strategy.

## Data Availability

The original contributions presented in the study are included in the article/supplementary material. Further inquiries can be directed to the corresponding author.
